# CAREGIVING CRISIS: NURSING ASSISTANT TRAINING REQUIREMENTS AND QUALITY OF LIFE IN SKILLED NURSING FACILITIES

**DOI:** 10.1093/geroni/igae098.0516

**Published:** 2024-12-31

**Authors:** Cassandra Barragan, Devlon Dean

**Affiliations:** Eastern Michigan University, Ypsilanti, Michigan, United States; Eastern Michigan University, Ypsilanti, Michigan, United States

## Abstract

Nursing Assistants (NAs) provide the majority of hands-on care for older adults in skilled nursing facilities (SNFs) while also providing companionship and psychological support. NAs receive a minimum of 75 hours of training in every state with 12 states requiring 120 or more (M=95.2). However, many NAs feel unprepared for the complexities of caring for older adults (Trinkoff et al., 2017). This lack of preparedness likely contributes to high staff turnover in SNFs (Gaivin, 2022) and directly impacts the quality of care (Castle & Endberg, 2006). This research explored the relationship between NA training hours and SNF quality of care citations in the United States. Data from the Centers for Medicare and Medicaid Services (CMS) SNF Quality Reporting Program (QRP) were used to determine the number of quality of life (QOL) citations for 5408 facilities in all 50 states and cross-referenced with the number of training hours required in each state. To control for the influence of a social worker, not required in smaller facilities, only facilities with 100 or more beds were examined. Regression analysis found for every hour increase in training hours, there will be an approximate 4% decrease in the predicted percentage of facilities with less than satisfactory QOL deficiencies. This research provides additional evidence that providing NAs with more training can improve QOL outcomes for residents of SNFs. This has implications for SNFs, assisted living, home health care, adult foster care, and other settings where NAs are employed.

